# Pre-exposure to *Candida albicans* induce trans-generational immune priming and gene expression of *Musca domestica*

**DOI:** 10.3389/fmicb.2022.902496

**Published:** 2022-09-27

**Authors:** Zhongxun Li, Lina Jia, Hong Yi, Guo Guo, Li Huang, Yingchun Zhang, Zhenlong Jiao, Jianwei Wu

**Affiliations:** ^1^School of Basic Medical Sciences, Guizhou Medical University, Guiyang, China; ^2^Department of Clinical Laboratory, The Second People’s Hospital of Yibin, West China Yibin Hospital, Yibin, China; ^3^Department of Pathology, The Affiliated Hospital of Southwest Medical University, Luzhou, China

**Keywords:** *Musca domestica*, *Candida albicans*, immune priming, phagosome, RNA sequencing

## Abstract

Insects have the phenomenon of immune priming by which they can have enhanced protection against reinfection with the same pathogen, and this immune protection can be passed on to their offspring, which is defined as “trans-generational immune priming (TGIP).” But whether housefly possesses TGIP is still unclear. Therefore, we used the housefly as the insect model and *Candida albicans* as the pathogen to explore whether the housefly is capable of eliciting TGIP, and RNA sequencing (RNA-seq) was performed to explore the molecular mechanism of TGIP of the housefly. We found that the housefly possesses TGIP, and adults pre-exposed to heat-killed *C. albicans* could confer protection to itself and its offspring upon reinfection with a lethal dose of *C. albicans*. RNA-seq results showed that 30 and 154 genes were differentially expressed after adults were primed with heat-killed *C. albicans* (CA-A) and after offspring larvae were challenged with a lethal dose of *C. albicans* (CA-CA-G), respectively. Among the differentially expressed genes (DEGs), there were 23 immune genes, including 6 pattern recognition receptors (PRRs), 7 immune effectors, and 10 immunoregulatory molecules. More importantly, multiple DEGs were involved in the Toll signaling pathway and phagosome signaling pathway, suggesting that the Toll signaling pathway and phagocytosis might play important roles in the process of TGIP of housefly to *C. albicans*. Our results expanded on previous studies and provided parameters for exploring the mechanism of TGIP.

## Introduction

Invertebrates only possess innate immunity (Melillo et al., 2018), so it had previously been thought that invertebrates did not have the phenomenon of immune memory. However, recent studies have found that the innate immune system of invertebrates also possesses a memory-like phenomenon, and the infection experience could confer protection against reinfection with the same pathogen, which is defined as immune priming in invertebrates (Milutinović and Kurtz, 2016; Meriggi et al., 2019). Many invertebrates can even confer protection to their offspring, which is defined as “trans-generational immune priming (TGIP)” (Tetreau et al., 2019). Insects are ideal models to investigate immune priming (Milutinović and Kurtz, 2016). It has been reported that many insects such as *Tenebrio molitor*, *Tribolium castaneum*, *Galleria mellonella*, *Drosophila melanogaster*, and mosquitoes possess immune priming, and many insects have TGIP (Christofi and Apidianakis, 2013; Vargas et al., 2016; Castro-Vargas et al., 2017; Futo et al., 2017; Taszłow et al., 2017; Tetreau et al., 2019).

The mechanism of insect immune priming is still not completely defined, and current studies suggested that both cellular immunity and humoral immunity might be involved in the process of immune priming (Contreras-Garduño et al., 2016). For example, the phagocytosis was enhanced in *D. melanogaster* immune primed with *Streptococcus pneumoniae* and *Pseudomonas aeruginosa* (Pham et al., 2007; Christofi and Apidianakis, 2013). In addition, the expression of antibacterial substances such as antimicrobial peptides (AMPs), lysozymes, and phenoloxidase (PO) was increased in the process of immune priming (Eleftherianos et al., 2006; Roth et al., 2010; Vargas et al., 2016). In many insects, females can transfer immune signals such as fragments of pathogens or immune effector mRNAs or proteins to their offspring; in addition, epigenetic modification may occur after parents were primed by pathogens, which might be the mechanisms of TGIP (Tetreau et al., 2019). However, the specific mechanism of these effects is still unclear. RNA-seq is an ideal tool to study the molecular mechanism of immune priming. Many transcriptomic studies have found that multiple immune genes and signaling pathways were involved in the process of immune priming, such as pattern recognition receptors (PRRs), AMPs, and Toll signaling pathway (Pham et al., 2007). However, there were differences in gene expression between different hosts to different pathogens (Zhao et al., 2013; Pinaud et al., 2016; Yi et al., 2019; Kulkarni et al., 2021; Sheehan et al., 2021). Therefore, the mechanism of invertebrate immune priming deserves further study.

The housefly *Musca domestica* (Diptera: Muscidae) is an ideal model for studying immune priming. The genome information of housefly showed that the gene diversity of immune signaling pathway is much higher than many other insects (Scott et al., 2014). As a result, the housefly may have a more effective immune response than many other insects. Many studies indicated that immune priming occurs in many dipteran insects, including *D. melanogaster* and mosquitoes (Milutinović and Kurtz, 2016). Our previous study has confirmed that housefly larvae have the phenomenon of immune priming (Li et al., 2022). However, whether the housefly possesses TGIP is still unclear. In this study, we used the housefly as the insect model and *C. albicans* as the pathogen to determine the TGIP of the housefly: (1) The TGIP effect of the housefly was verified; (2) RNA-seq was performed to search the genes and signaling pathways which might take part in the process of TGIP of the housefly. Our results showed that housefly adults primed with heat-killed *C. albicans* could confer protection to themselves and their offspring upon reinfection with a lethal dose of *C. albicans*. A large number of differentially expressed genes (DEGs) were identified, and more importantly, multiple DEGs were involved in the Toll signaling pathway and phagosome signaling pathway, suggesting that the Toll signaling pathway and phagocytosis may play a part in the process of TGIP of the housefly. Our results expanded on previous studies and provided parameters for exploring the mechanism of TGIP.

## Materials and methods

### Housefly rearing

The housefly (*M. domestica*) was reared at the Department of Parasitology, Guizhou Medical University (Guiyang, China) as described previously (Wang et al., 2016). Houseflies were raised at 26 ± 1°C with a relative humidity of 70–80% and a photoperiod of 12 h light/12 h dark cycle. Housefly adults were fed with a mixture of sugar and milk powder at a ratio of 1:1, and distilled water was supplied for drinking. Housefly larvae were raised on an artificial diet comprising wheat bran and water.

### Fungal culturing

*Candida albicans* (ATCC10231) was inoculated in liquid Sabouraud dextrose broth (SDB) and cultivated at 37°C for 14 h with agitation to the logarithmic growth phase (Wang et al., 2017). Fungal cells were harvested by centrifugation at 3,000 rpm for 5 min and washed three times with sterile phosphate-buffered saline (PBS), and resuspended in PBS. Fungal cells were heat killed at 95°C for 15 min (Sheehan et al., 2021). Heat-killed *C. albicans* was used to prime housefly adults, as heat-killed *C. albicans* would elicit an immune response without the direct cost of infection.

### Determination of the priming and challenge dose

Housefly adults were injected with 210 nl of suspension at the center of the dorsal using the Nanoliter Injector (Drummond Scientific Co., United States) for pre-exposure (priming) and reinfection (challenge) (Xiu et al., 2016). To determine the lethal dose of housefly adults, 3 days old adults (*n* = 50, male: female = 1: 1) were injected with *C. albicans* at the concentration of 1 × 10^8^ CFU/ml, 2 × 10^8^ CFU/ml, or 3 × 10^8^ CFU/ml, respectively; and PBS was injected as control. Mortality of adults was recorded daily for 7 days after infection.

Heat-killed *C. albicans* was used for priming. To determine the appropriate priming dose of the housefly, 3 days old adults (*n* = 80, male: female = 1: 1) were first primed with heat-killed *C. albicans* at the concentration of 1 × 10^7^ CFU/ml or 1 × 10^8^ CFU/ml, respectively, and PBS was injected as control. At 48 h after priming, adults in each group (*n* = 50, male: female = 1: 1) were injected with a lethal dose of *C. albicans* for the challenge. Mortality of adults was recorded daily for 7 days after the challenge.

The experiments were repeated three times independently. Survival analyses were performed using the Log-rank test of the Kaplan-Meier survival analysis (Christofi and Apidianakis, 2013).

### Immune priming and challenge

**FIGURE 1:**
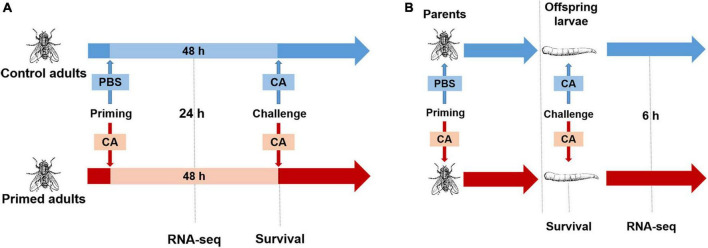
Experimental design. **(A)** Within generation immune priming. Housefly adults were primed by injection of heat-killed *C. albicans* or PBS as the control. Samples were collected at 24 h post-priming for RNA-seq. At 48 h after priming, adults were challenged with a lethal dose of *C. albicans*, and the survival was monitored after the challenge. **(B)** Trans-generational immune priming. Parental adults were primed by injection of heat-killed *C. albicans* or PBS as control, then mated and laid eggs, when offspring reached the third instar, larvae were challenged with a lethal dose of *C. albicans*, survival was monitored after the challenge, and samples were collected at 6 h post-challenge for RNA-seq.

First, we explored the protection effect for adults (within generation immune priming). Housefly adults (*n* = 100, male: female = 1: 1) were primed, and controls were injected with PBS or left naive. At 48 h post-priming, adults in each group (*n* = 80, male: female = 1: 1) were challenged with *C. albicans*. Mortality of adults was recorded daily for 7 days after the challenge (Figure 1A).

Second, we explored the protection effect for offspring larvae (TGIP). Virgin adults (*n* = 100, male: female = 1: 1) were primed, and controls were injected with PBS. Then, adults in each group were mated immediately after priming, and females laid eggs 2–3 days after priming. When eggs developed into the third instar (3 days after eggs laid), larvae (*n* = 80) were challenged with *C. albicans*. Mortality of larvae was detected at 24 h post-challenge (Figure 1B; Castro-Vargas et al., 2017).

The experiments were repeated three times independently (*n* = 80/group in each replicate). Survival analyses were performed using the Log-rank test of the Kaplan-Meier survival analysis (Christofi and Apidianakis, 2013).

### Sample collection of transcriptomic analysis

Housefly virgin adults (male: female = 1: 1) were primed, and controls were injected with PBS. At 24 h post-priming, adults were collected for RNA-seq. A library was comprised of 1 male and 1 female adults, and there were 3 replicates in each group, making 6 libraries in total. *C. albicans* primed and PBS control adults were denoted as CA-A and PBS-A, respectively (Figure 1A).

Offspring larvae from primed and control parents were challenged with *C. albicans*. At 6 h post-challenge, larvae were collected for RNA-seq. A library comprised of 2 larvae, and there were 3 replicates in each group, also making 6 libraries in total. *C. albicans* primed offspring larvae and PBS control offspring larvae were denoted as CA-CA-G and PBS-CA-G, respectively (Figure 1B).

### Total ribonucleic acid extraction

Total ribonucleic acid (RNA) was extracted using TRIzol reagent (Invitrogen, United States) following the manufacturer’s protocol. Total RNA samples were incubated with RNase-free DNase I to eliminate contaminating DNA. RNA concentration was measured using a NanoDrop 2000 spectrophotometer (Thermo Fisher Scientific, United States).

### Ribonucleic acid sequencing and analysis

RNA-seq was performed by Beijing Genomics Institute (BGI) using DNBSEQ platform, and 1 μg of total RNA was used to establish cDNA library. First, the mRNA with poly A tail was enriched by magnetic beads with Oligo dT. Then, the mRNA was interrupted into RNA fragments, and the second strand of cDNA was synthesized to form double-stranded DNA. The double-stranded DNA was connected to a connector and amplified by PCR with specific primers. PCR products were thermally denatured into single strands and formed the single strand circular DNA library with a bridge primer, and then sequenced.

Raw reads were filtered to obtain clean reads, which were compared with *M. domestica* genome (*Musca_domestica*-2.0.2) by TopHat2 software (Kim et al., 2015). The identification and counting of DEGs were conducted with RSEM software (Li and Dewey, 2011), and the corrected *P*-value < 0.05 was set as the threshold for significantly differential expression. The DEGs analysis was performed using gene ontology (GO) for the classification of predicted genes, as well as the Kyoto encyclopedia of genes and genomes (KEGG), to analyze the related signaling pathways (Xu et al., 2019).

## Results

### Priming and challenge dose

**FIGURE 2:**
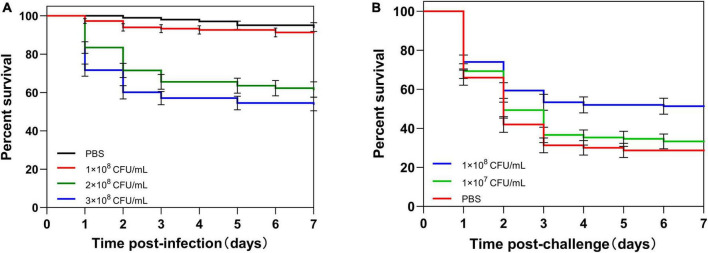
Survival of housefly adults after infection. **(A)** Adults were injected with different concentrations (1 × 10^8^, 2 × 10^8^, or 3 × 10^8^ CFU/ml) of *C. albicans* or PBS. **(B)** Adults were first primed with different concentrations (1 × 10^7^ or 1 × 10^8^ CFU/ml) of heat-killed *C. albicans* or PBS, then challenged with a lethal dose of *C. albicans.* The survival data of each condition were generated from three replicates, and each had 50 adults. The survival difference was compared using the Log-rank test of the Kaplan-Meier survival analysis. The results are reported as mean ± SE from three independent experiments.

To determine the lethal dose of housefly adults, adults were injected with different concentrations of *C. albicans*. About 90% of adults survived after the injection of 1 × 10^8^ CFU/ml *C. albicans* (non-lethal dosage). However, the injection of 3 × 10^8^CFU/ml *C. albicans* caused the death of approximately 70% of the injected adults after 7 days. As a result, we used 3 × 10^8^ CFU/ml *C. albicans* as the lethal challenge dose of housefly adults (Figure 2A).

To determine the appropriate priming dose of the housefly, adults were primed by injection of different concentrations of heat-killed *C. albicans*, and then, adults were challenged with a lethal dose of *C. albicans* at 48 h post-priming. There was no significant difference in survival rate between 1 × 10^7^ CFU/ml *C. albicans* primed larvae and control larvae (*P* = 0.37). However, the survival of 1 × 10^8^ CFU/ml primed larvae was higher than the control larvae (*P* < 0.001) (Figure 2B). These results indicated that housefly adults injected with 210 nl of heat-killed *C. albicans* at the concentration of 1 × 10^8^ CFU/ml could induce immune priming, and this priming dose could be used for further investigations.

### Within and trans-generational immune priming

**FIGURE 3:**
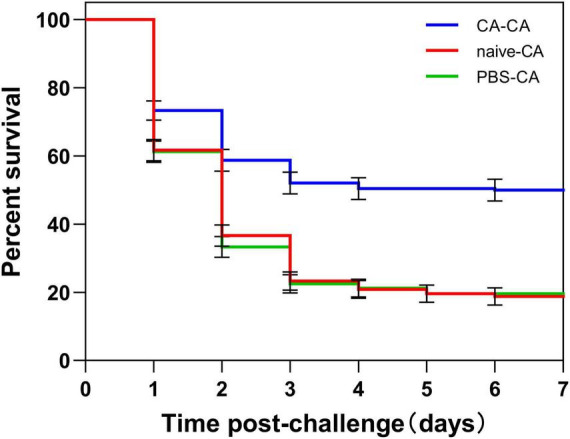
Survival of housefly adults after challenge. Adults were first primed by injection of heat-killed *C. albicans* (CA) or injection of PBS as injury control (PBS), or left untreated (naive), then adults challenged with a lethal dose of *C. albicans.* The survival data of each condition were generated from three replicates, and each had 80 adults. The survival difference was compared using the Log-rank test of the Kaplan-Meier survival analysis. The results are reported as mean ± SE from three independent experiments.

**FIGURE 4:**
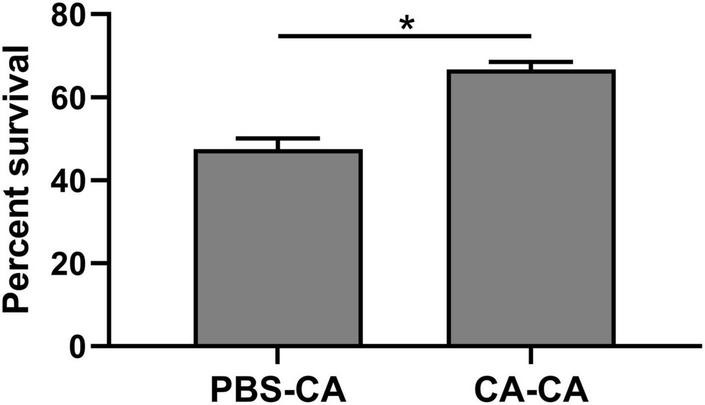
Survival of offspring larvae 24 h post-challenge. Parental adults were first primed by injection of heat-killed *C. albicans* (CA) or injection of PBS as injury control (PBS), and offspring larvae were challenged with a lethal dose of *C. albicans.* The survival data of each condition were generated from three replicates, and each had 80 larvae. The survival difference was compared using the Log-rank test of the Kaplan-Meier survival analysis. The results are reported as mean ± SE from 3 independent experiments. Asterisk indicates significant differences between priming treatment and PBS control (*P < 0.05).

First, housefly adults were primed with heat-killed *C. albicans*. We then detected the within-generation immune priming of the housefly, and primed adults were challenged with a lethal dose of *C. albicans* at 48 h post-priming. The survival of *C. albicans* primed adults was significantly higher than that of the naive adults (*P* < 0.0001) and PBS control adults (*P* < 0.0001), but there was no significant difference in survival rate between naive and PBS control adults (*P* = 0.39) (Figure 3). Then, we detected the TGIP of the housefly, offspring larvae were challenged with a lethal dose of *C. albicans*, and the survival of *C. albicans* primed offspring larvae was significantly higher than PBS control (*P* < 0.05) (Figure 4). The results indicated that housefly adults primed with heat-killed *C. albicans* could induce within and TGIP.

### Transcriptomic response after priming and challenge

To identify the systemic transcriptomic response of housefly TGIP, we conducted RNA-seq to compare transcriptomes after adults were primed with heat-killed *C. albicans* and offspring larvae challenged with a lethal dose of *C. albicans*. A total of 12 libraries were conducted.

#### Overview of transcriptomic response after priming and challenge

**FIGURE 5:**
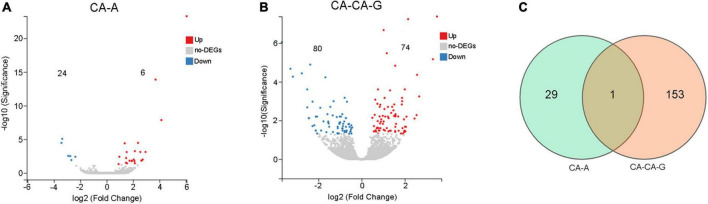
The number of differentially expressed genes. **(A)** The volcano plots of the DEGs in CA-A. Significant differentially expressed genes were treated with red dots (upregulated) or blue dots (downregulated). **(B)** The volcano plots of the DEGs in CA-CA-G. Significant differentially expressed genes were treated with red dots (upregulated) or blue dots (downregulated). **(C)** Venn diagram of differentially expressed genes. The number in each circle represents the total number of DEGs in each sample, and the overlapping part of the circles indicates that the DEG is shared in both samples.

A total of 30 DEGs were identified after adults were primed with heat-killed *C. albicans* (CA-A), 24 upregulated and 6 downregulated (Figure 5A), among which 8 were immune genes (Table 1). A total of 154 DEGs were identified after offspring larvae were challenged with a lethal dose of *C. albicans* (CA-CA-G), 80 upregulated and 74 downregulated (Figure 5B), among which 16 were immune genes (Table 2). There was 1 shared DEGs in CA-A and CA-CA-G (Figure 5C), which was a male accessory gland serine protease inhibitor, a serpin family immunoregulatory molecule, and all were upregulated in both groups.

**TABLE 1:** Expression of differentially expressed immune genes in CA-A.

Gene ID	Description	Gene expression (FPKM)	
		PBS-A	CA-A
**PRRs**			
101890291	Lectin subunit alpha	9.456	25.51
101899192	Lectin subunit alpha isoform X2	2.75	16.213
**Immune effectors**			
101892478	Lysozyme B-like	86.526	10.226
105261502	Lysozyme 1-like	290.626	1053.333
**Immunoregulatory molecules**			
101896495	Serine protease SP24D-like	14.363	58.026
101901662	Serine protease SP24D-like	14.363	58.026
101900762	Serine proteases 1/2-like	0	39.316
101892327	Male accessory gland serine protease inhibitor	1.25	39.713

**TABLE 2:** Expression of differentially expressed immune genes in CA-CA-G.

Gene ID	Description	Gene expression (FPKM)	
		PBS-CA-G	CA-CA-G
**PRRs**			
101898821	Fibrinogen C domain-containing protein 1 isoform X1	175.496	348.826
101896844	Fibrinogen-like protein 1	118.023	69.003
101888722	Gram-negative bacteria-binding protein 2 isoform X1	25.33	45.056
101893128	Lectin subunit alpha	1459.393	4866.356
**Immune effectors**			
101889632	Sarcotoxin-1B	84.8	480.016
101888757	Sarcotoxin-1C	32.656	223.153
101889972	Sarcotoxin-1D	0.836	32.51
101897990	Heat shock protein 23	6.676	1.943
101888337	10 kDa heat shock protein, mitochondrial	66.51	31.18
**Immunoregulatory molecules**			
101900180	Serine protease easter isoform X3	226.766	328.786
101901013	Serine protease Hayan-like	15.633	23.333
101895989	Serine protease inhibitor 77Ba	31.803	59.813
101888327	Serine protease persephone	23.04	41.463
101898345	Serine protease SP24D	25.876	10.896
101892493	Male accessory gland serine protease inhibitor	44.323	6.7
101892327	Male accessory gland serine protease inhibitor	104.866	346.8

#### Gene ontology and Kyoto encyclopedia of genes and genomes enrichment

**FIGURE 6:**
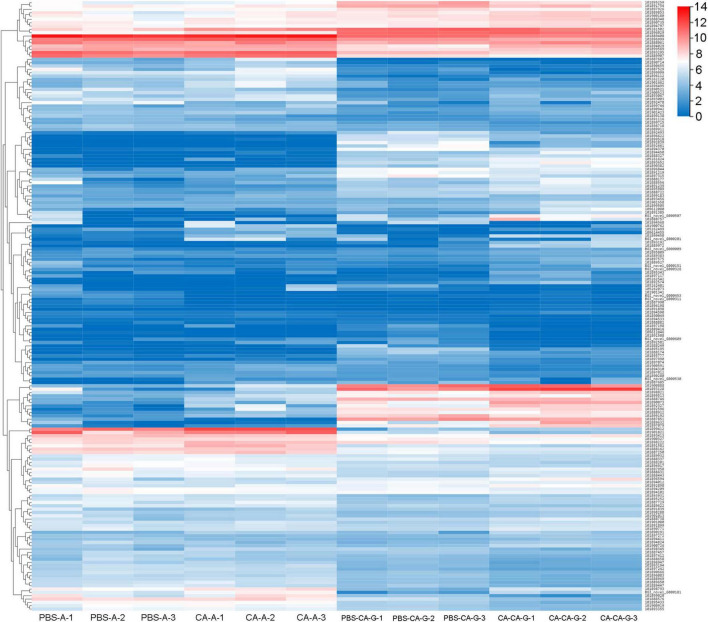
Clusters of the differentially expressed genes. Heat map shows expression kinetics of the 183 transcripts of housefly adults primed with *C. albicans* or injected with PBS as control and offspring larvae challenged with a lethal dose of *C. albicans*. The color scale indicates the expression level of the transcripts.

**FIGURE 7:**
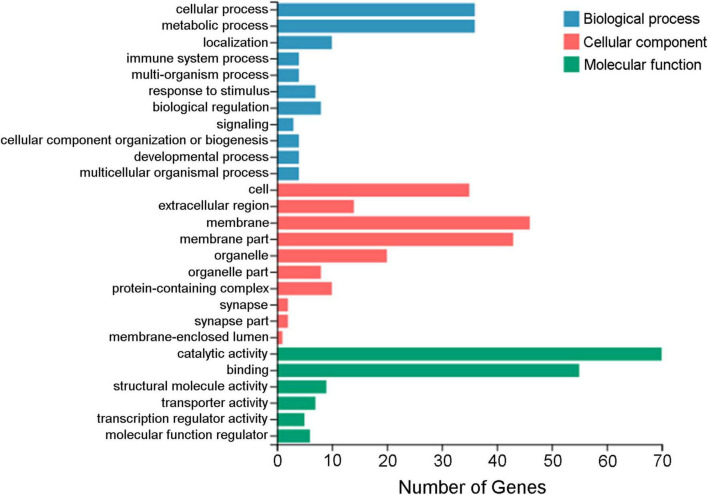
GO annotation of the differentially expressed genes. The *x*-axis indicates the number of genes, and the *y*-axis indicates the subcategories.

**FIGURE 8:**
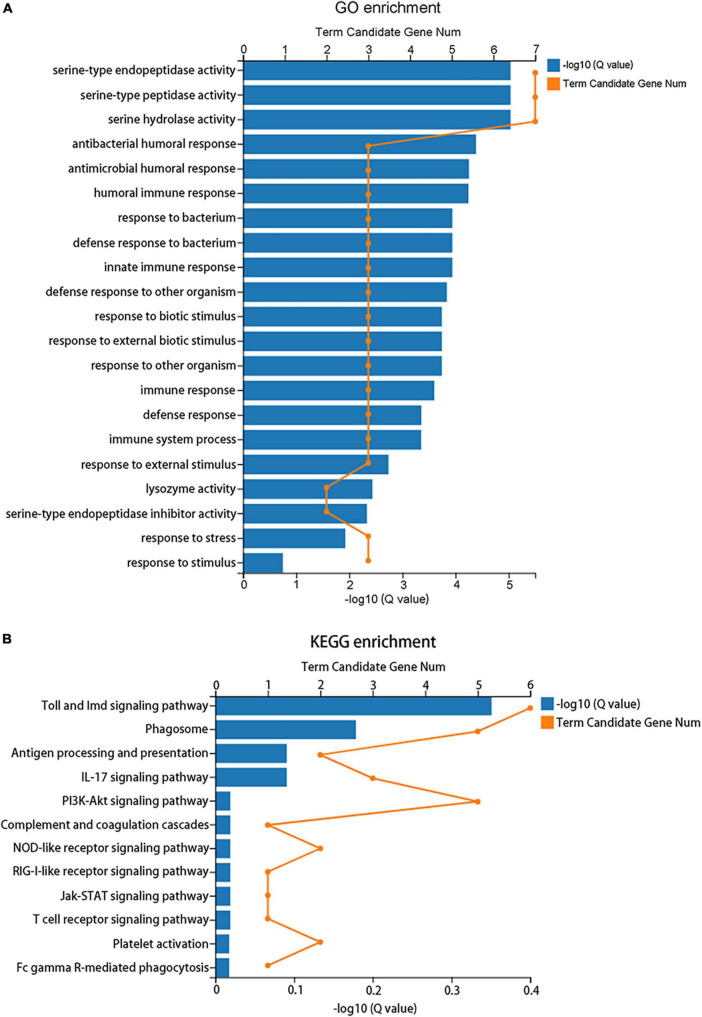
Enrichment analysis of the differentially expressed genes. **(A)** GO enrichment indicates that 24 DEGs were enriched into 21 immune-related GO terms. **(B)** KEGG enrichment indicates that 25 DEGs were enriched into 12 immune signaling pathways.

A total of 183 DEGs were identified in this study (Figure 6). GO function annotation showed that the DEGs were mainly involved in 3 categories, namely, biological process, cellular component, and molecular function, among which multiple DEGs were involved in immune system processes and metabolic processes (Figure 7). GO enrichment showed that the DEGs were enriched into 899 GO terms (Supplementary Table 1), among which 24 DEGs were enriched into 21 immune-related GO terms (Figure 8A). KEGG enrichment showed that the DEGs were enriched into 108 signaling pathways (Supplementary Table 2), among which 25 DEGs were enriched into 12 immune signaling pathways (Figure 8B).

#### Immune genes and signaling pathways

**FIGURE 9:**
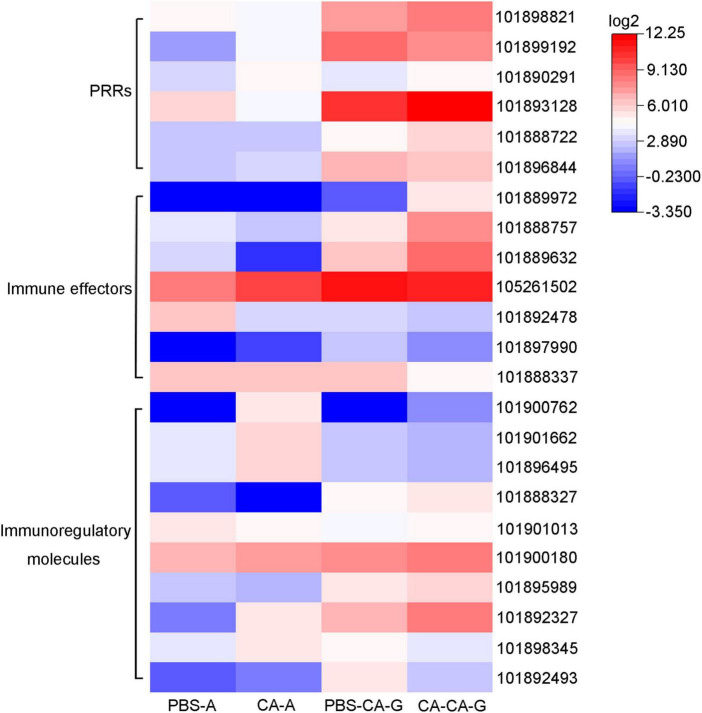
Expression of differentially expressed immune genes. Heat map shows the expression of differentially expressed immune genes, including 6 PRRs, 7 immune effectors, and 10 immunoregulatory molecules. The color scale indicates the expression level of the transcripts.

Immune system plays a key role in the process of immune priming, so we focused on immune genes and immune signaling pathways. A total of 23 differentially expressed immune genes were identified, including 6 PRRs, 7 immune effectors, and 10 immunoregulatory molecules (Figure 9). There were 6 DEGs involved in the Toll and Imd signaling pathways, namely, serine protease persephone (LOC101888327), serine protease easter isoform X3 (LOC101900180), serine protease Hayan-like (LOC101901013), GNBP2 isoform X1 (LOC101888722), uncharacterized LOC105262073, and uncharacterized LOC105262401; and there were 5 DEGs involved in the phagosome signaling pathway, namely, lectin subunit alpha (LOC101890291), lectin subunit alpha (LOC101893128), lectin subunit alpha isoform X2 (LOC101899192), crustapain (LOC101888443), and hypothetical protein LOC101888348. In addition, there were multiple genes involved in another 10 immune signaling pathways (Figure 8B).

#### Metabolic genes

Except for immune genes, there were multiple DEGs involved in the metabolic process. There were 6 DEGs involved in the metabolic process in CA-A (Supplementary Table 3) and 24 DEGs involved in the metabolic process in CA-CA-G (Supplementary Table 4). These metabolic genes were involved in a variety of metabolic processes, such as steroid biosynthesis, cholesterol metabolism, and glycolysis/gluconeogenesis process, which can provide energy for immune response.

## Discussion

Insects have the phenomenon of immune priming by which they can have enhanced protection against reinfection with the same pathogen that has infected previously (Milutinović and Kurtz, 2016; Meriggi et al., 2019). The protection of immune priming can last for a long time and even be passed on to their offspring, which is defined as “TGIP” (Tetreau et al., 2019). Whether there is TGIP in housefly is still unclear. Therefore, we used the housefly as the insect model and *C. albicans* as the pathogen to study the TGIP of the housefly, and RNA-seq was performed to explore the molecular mechanism of TGIP in housefly to *C. albicans*.

In this study, we found that housefly have the phenomenon of TGIP, adults primed by injection of heat-killed *C. albicans* could confer protection for itself and its offspring upon reinfection with a lethal dose of *C. albicans.* Recent studies on immune priming have made great progress, and it has been found that *T. molitor*, *T. castaneum*, *Bombus terrestris*, *Apis mellifera*, and many other insects possess TGIP (Sadd and Schmid-Hempel, 2009; Roth et al., 2010; Tidbury et al., 2011; Salmela et al., 2015; Dhinaut et al., 2018); however, the mechanism of TGIP is still not completely defined.

RNA-seq was commonly used to study the molecular mechanism of insect immune priming, by which to detect the expression levels of genes and to identify genes and signaling pathways that may be involved in immune priming (Wang et al., 2009; Vargas et al., 2016). Recent studies indicated that there were certain differences in the gene expression during immune priming of insects to different pathogens. The most identified genes during immune priming were a variety of PRRs and immune effectors (Zhao et al., 2013; Greenwood et al., 2017; Tate et al., 2017; Yi et al., 2019; Kulkarni et al., 2021; Maya-Maldonado et al., 2021). To explore the molecular mechanism of TGIP of the housefly to *C. albicans*, RNA-seq was performed to detect the gene expression after immune priming. To explore the duration of immune effects after priming of adults, we collected adults at 24 h after priming; and to explore the immune response at the early stage of infection, we collected offspring larvae at 6 h after the challenge.

The first step to start immune responses is PRRs to recognize pathogen-associated molecular patterns (PAMPs), which in turn activate immune signaling pathways and produce immune effects, and this may be the key step in immune priming (Schmid-Hempel, 2005; Sułek et al., 2021). It has been found that the innate immune system of insects has a variety of PRRs (Kim et al., 2000; Zaidman-Rémy et al., 2011; Meiers et al., 2019; Li, 2021). In this study, many PRRs were differentially expressed. Lectins act as ligands to recognize polysaccharides, lipopolysaccharides, or peptidoglycan components of bacteria or fungi (Meiers et al., 2019). Many studies indicated that the expression of lectins was changed during immune priming of *Helicoverpa armigera*, *T. castaneum*, and *Biomphalaria glabrata* (Zhao et al., 2013; Pinaud et al., 2016; Greenwood et al., 2017). Invertebrate fibrinogen-related proteins (FREPs) show that lectin activity, especially fibrinogen C domain-containing protein 1, plays an important role in the recognition of fungi (Pilecki and Moeller, 2020). In addition, fibrinogen can form fibrin matrices, from which to prevent the spread of pathogens (Ko and Flick, 2016). Pinaud et al. (2016) found that the expression of FREPs in *B. glabrata* was changed after immune primed with *S. mansoni.* Gram-negative bacteria-binding proteins (GNBPs) can recognize the glucan of fungi and initiate immune responses (Matskevich et al., 2010). Recent studies indicated that the expression of GNBPs was changed during immune priming of *Anopheles gambiae* and *T. castaneum* (Tate et al., 2017; Kulkarni et al., 2021). Collectively, the above PRRs might play important roles in the process of TGIP of the housefly.

After PRRs recognize PAMPs, the immune signaling pathways are activated, and many immune effectors are induced, such as AMPs and lysozymes (Morozova et al., 2020; Sułek et al., 2021). In this study, multiple immune effectors were differentially expressed. Lysozymes are broad-spectrum antibacterial proteins and can exert antifungal activity (Sowa-Jasiłek et al., 2016). Recent studies indicated that the expression of lysozymes was changed during immune priming in *Bombyx mori*, *H. armigera*, *Mytilus galloprovincialis*, *T. castaneum*, and *A. gambiae* (Zhao et al., 2013; Greenwood et al., 2017; Rey-Campos et al., 2019; Yi et al., 2019; Kulkarni et al., 2021). Heat shock proteins (HSPs) are relatively conservative proteins that can participate in various biological processes including immunity (Bolhassani and Agi, 2019). Many studies indicated that the expression of HSPs was changed during immune priming in *B. mori*, *H. armigera*, *M. galloprovincialis*, and *B. glabrata* (Zhao et al., 2013; Pinaud et al., 2016; Rey-Campos et al., 2019; Yi et al., 2019). Interestingly, unlike previous studies, three sarcotoxin genes were differentially expressed in this study. However, the expression of sarcotoxin was not affected in previous immune priming studies. Sarcotoxin is a cecropin family AMP, which can exert antibacterial activity (Elhag et al., 2017). The possible reason is that the pathogens used in these studies were bacteria or parasites, while *C. albicans* was a fungus, so it is speculated that sarcotoxin may play an important role in the process of immune priming of insects against fungi.

Among the DEGs identified in this study, many genes were enriched into immune signaling pathways, among which several genes were enriched into the Toll signaling pathway. As the Toll signaling pathway was involved in gram-positive bacteria and fungi infection in insects (Hanson and Lemaitre, 2020), it is suggested that the Toll signaling pathway may play a part in the immune priming of the housefly to *C. albicans*, and this is consistent with the discovery that Toll signaling pathway was involved in immune priming of *D. melanogaster* to *S. pneumoniae*. In addition, 5 genes were enriched into the phagosome signaling pathway. Previous studies indicated that phagocytosis plays an important role during immune priming of *D. melanogaster* (Pham et al., 2007; Christofi and Apidianakis, 2013), so it is speculated that phagocytosis may also play a part in immune priming of the housefly.

Immune responses are complex processes that need to be precisely regulated, so there are many immunoregulatory molecules involved in the process of immunity, among which serine proteases and serine protease inhibitors (Serpins) play important regulatory roles in insects (Meekins et al., 2017). Previous studies indicated that the expression of many serine proteases and Serpins was changed during immune priming (Pinaud et al., 2016; Yi et al., 2019). In this study, multiple serine proteases and Serpins were also differentially expressed, suggesting that serine proteases and Serpins may play regulatory roles during the immune priming of the housefly. In addition, the metabolism is affected during immune response as it requires energy (Dionne, 2014). The expression of multiple metabolic genes was differentially expressed during immune priming of the housefly, but it is not clear how metabolism is involved in immune priming. As a result, further research is needed to determine how these genes are involved in the process of immune priming of the housefly.

In conclusion, we used *C. albicans* as the pathogen to study the TGIP of the housefly. Our results showed that the housefly possesses TGIP, and the expression of multiple genes including immune genes and metabolic genes was changed during immune priming. More importantly, multiple DEGs were involved in the Toll signaling pathway and phagosome signaling pathway, suggesting that the Toll signaling pathway and phagocytosis may play important roles in the process of TGIP of the housefly. Our results expanded on previous studies and provided parameters for exploring the mechanism of immune priming.

## Data availability statement

The data presented in this study are deposited in the NCBI repository (https://www.ncbi.nlm.nih.gov/), accession number, PRJNA781280.

## Author contributions

ZL and JW designed the project and wrote the manuscript. ZL, LJ, LH, and YZ carried out the experiments. ZL, HY, and ZJ analyzed the data. GG, ZJ, and JW reviewed the manuscript. All authors contributed to the article and approved the submitted version.
